# Numerical and Experimental Investigation of Parameters in Cement Delivery Through Spinal Implants

**DOI:** 10.3390/ma18245566

**Published:** 2025-12-11

**Authors:** Damian Obidowski, Lechosław F. Ciupik, Agnieszka Kierzkowska, Piotr Reorowicz, Artur Bonik, Zbigniew Tyfa, Krzysztof Sobczak, Edward Słoński, Krzysztof Jóźwik

**Affiliations:** 1 Institute of Turbomachinery, Lodz University of Technology, 90-924 Łódz, Poland; piotr.reorowicz@p.lodz.pl (P.R.); zbigniew.tyfa@p.lodz.pl (Z.T.); krzysztof.sobczak@p.lodz.pl (K.S.); krzysztof.jozwik@p.lodz.pl (K.J.); 2 LfC sp. z o.o., Kożuchowska 41, 65-364 Zielona Góra, Poland; lfc@lfc.com.pl (L.F.C.); a.kierzkowska@lfc.com.pl (A.K.); a.bonik@lfc.com.pl (A.B.); e.slonski@lfc.com.pl (E.S.)

**Keywords:** bone cement delivery, spinal implant design, Computational Fluid Dynamics, bone cement rheological properties, vertebroplasty simulation

## Abstract

Bone cement is used in spinal procedures and can be used alone or in combination with an implant to stabilize spine and relieve pain. Despite benefits, complications remain a concern. This study investigates how the internal geometry of a spinal implant device affects injection pressure and cement distribution. Two design groups (G1 and G2), differing in lateral channel angle, were analyzed across three functional variants using CFD (Computational Fluid Dynamics) simulations. CFD modeling employed a two-phase (air–cement) flow. Experimental tests confirmed simulation tests and revealed that angled channels (G2) promoted more uniform cement flow. CFD analysis showed reduced pressure on the syringe plunger, especially when the central channel was blocked. Threaded configurations increased the needed pressure but had minimal impact on flow distribution. G2 required a higher force exerted on the syringe plunger than G1. The study concludes that channel geometry significantly affects the required cement delivery pressure and implant fixation, which translates into the implant–bone interface. While certain configurations improve flow uniformity, elevated injection pressure may pose risks. These findings support optimizing implant design and cement delivery techniques, contributing to safer and more effective implant-based spinal surgeries with bone cement augmentation.

## 1. Introduction

Bone cement is a material that is frequently used in surgical interventions for spinal diseases. The utilization of this material is most frequently observed in the context of vertebroplasty [1], kyphoplasty [2], stentoplasty [3], and analogous techniques that necessitate vertebral augmentation. Its application encompasses minimally invasive treatment of compression fractures of the spine caused by osteoporosis [4,5], the filling of neoplastic lesions [6], revision procedures, and instances where bone cement is required to strengthen weakened bone structures [7,8].

Bone cement is a material that exhibits a number of noteworthy properties, which impact the course of the procedure and its interaction with the patient’s body. In this publication, particular attention is paid to the rheological properties of bone cement, which have a significant impact on the technique of its administration and the appropriate surgical instruments [9]. In applying bone cement, a physician faces a twofold challenge: achieving optimal penetration of the trabecular bone of the vertebrae while ensuring that the cement does not leak outside the vertebral body, as this may lead to postoperative complications [9,10]. In the interest of patient safety, it is imperative to accurately determine the rheological properties of the administered preparation, along with any alterations that may occur over time. The manner in which the properties of the cement undergo change over time constitutes a critical parameter that is given full consideration during the planning stage of the procedure.

The utilization of cement in spinal bone procedures confers a multitude of advantages, including immediate analgesia [6], mechanical stabilization of the bone structure and restoration of load-bearing capacity, prevention of further damage [11], and a reduced hospital stay for the patient. Cementoplasty procedures are frequently performed under local anesthesia, a method that is adequate for patients suffering from additional diseases, elderly patients, and high-risk patients [6,12]. Notwithstanding the numerous advantages associated with the utilization of this biomaterial in clinical practice, there are a number of risks associated with its use.

The most prevalent adverse events include cement leaks outside the bone bed, which, according to literature, occur clinically in 5–10% of cases, while radiographic studies reveal leaks in as many as 80% of patients [13,14]. The underlying causes of such occurrences are hypothesized to be excessive injection pressure, excessive cement volume, or damage to the shaft walls. Despite the elevated risk of leakage, adverse symptoms are not typically observed. It is noteworthy that only a minute fraction of cases—1%—result in severe complications, or, in exceptional cases, result in the mortality of the patient. The most frequently observed complications include pulmonary embolism, cerebral embolism, renal artery embolism, pulmonary hypertension, acute respiratory distress syndrome, and heart failure [6,11,15].

Furthermore, inconsistencies have been identified with regard to the biomechanical aspects of cement utilization in spinal applications. It has been suggested by certain authors that the rigidity of bone cement can result in secondary fractures of adjacent vertebrae [16]. It has been demonstrated by other researchers that the presence of cement does not have a significant impact on the formation of new fractures [4]. A significant factor in mitigating the risk of cement leakage during spinal surgery is the implementation of an appropriate surgical technique, encompassing the method of cement delivery and the injection parameters associated with its application. This underscores the necessity to enhance the cement administration technique and automate this step of the procedure [8].

In the relevant literature, particular emphasis is placed on the time-varying nature of bone cement viscosity. Two-component cements in powder-liquid form undergo polymerization after mixing. In the initial phase, they remain fluid; in the application phase, they reach a dough-like consistency, as specified in the manufacturer’s instructions for use of PMMA cements [17,18]. Ultimately, they harden into a solid material [19,20,21]. Low-viscosity cements have been shown to provide adequate penetration into trabecular bone; however, their injection through wider cannulas has been associated with an increased risk of leakage [19,20,21]. This risk increases further with increasing injection pressure, which may promote uncontrolled cement discharge [19,22]. Standard and medium-viscosity cements exhibit reduced working times and are generally applied manually through cannulas, with the transpedicular approach being the most common for reasons of safety [19,20,22,23]. It is evident that high-viscosity formulations have the capacity to reduce the risk of leakage. However, it is important to note that they require higher injection pressure, which is widely considered to be their primary disadvantage [19,21,22]. An interesting group of experimental cements, in which extended working time and reduced exothermic reaction have been achieved by rendering the negative changes in viscosity and injection technique [24]. A detailed comparison of the properties of the individual formulations is presented in Table 1.

In this study, the CFD results are intended to validate geometry-driven flow effects primarily within the clinically relevant workable phase of PMMA (pre-gel/early-gel), i.e., before the rapid post-gel rise in viscosity. The assumption of constant viscosity is therefore limited to this early injection window.

The objective of the present study was to assess the impact of the geometry of the fluid delivery channels located within the spinal device on the injection pressure and the volume of cement delivered to the immediate vicinity of the device. The assessments were conducted through numerical studies and subsequently supported by empirical laboratory investigations.

## 2. Materials and Methods

### 2.1. Geometric Description of the Models Studied

**Figure 1:**
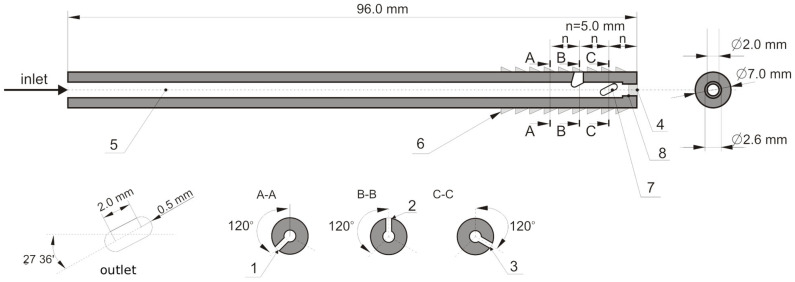
Diagram of the implant device: 1–4—outlet holes (4 blocked if desired), 5—central feed channel, 6—thread (optional), 7—outline of the side feed channel shape, 8—central hole blocker (optional).

The study incorporated an implant model with a network of internal channels for medium delivery consisting of a central channel corresponding to a standard cannula for bone cement delivery and a set of three lateral channels (Figure 1). The inlet cross-section of the cannula through which the bone cement is delivered has an area of 5.3 mm^2^. The area of each of the side outlet openings (1 ÷ 3) was designed to be approximately 2.8 mm^2^, while the area of the central outlet opening (4) was determined to be 3.5 mm^2^. The total area of the outlet openings was found to be approximately 11.8 mm^2^.

The configuration of the outlet holes has been engineered in a manner that, should one or more apertures become occluded, ensures the outflow of bone cement, thereby minimizing the risk of the implant failing, and allows adequate fixation within the bone structure.

**Figure 2:**
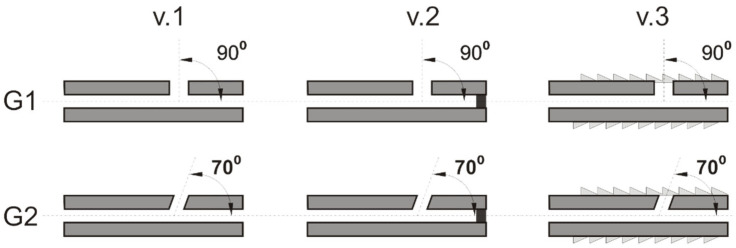
Variants of channel configurations in the implant device under investigation.

The study employed implant models with a two-stage central channel, measuring a total of 96 mm in length, with an initial diameter of 2.6 mm spanning a length of 94 mm, and a final diameter of 2.1 mm over a length of 2 mm. The variant designs were divided into two groups, designated G1 and G2. In group G1, the lateral delivery channels were oriented perpendicularly (90°) to the central channel, while in group G2, they were oriented at an angle of 70° relative to the central channel (Figure 2). In each of the G1 and G2 design groups, the following three variants were utilized:

- v.1 variant with no external thread in the vicinity of the outlet holes (1, 2, 3) and the presence of unblocked outlet holes (1, 2, 3, 4).
- v.2 variant with no external thread in the vicinity of the outlet holes (1, 2, 3) and the presence of a blocked central outlet hole (4).
- v.3 version is characterized by the presence of an external thread, which facilitates its implantation into bone. Additionally, all outlet holes (1, 2, 3, 4) are open.

Research on cement flow in the described channels of the device was carried out using numerical modeling and experimental tests. It is evident that a substantial proportion of the research devoted to the selection of appropriate geometric parameters of the channels was undertaken utilizing Computational Fluid Dynamics (CFD) methods. This was due to the high cost of the material, the costs of preparing subsequent channel variants and their disposal, as well as the requirements for special conditions recommended for cement feeding. Subsequently, a selection of device variants was manufactured and subjected to experimental testing in order to support the findings of numerical analyses.

In order to validate the numerical studies, in both approaches, the pressure, feeding force, and amount of medium discharging through the outlet holes were evaluated. A series of numerical studies was carried out for all groups (G1, G2) and solution variants (v.1, v.2, v.3). An experimental investigation was conducted on models G1 and G2 in functional variant v.3. The experimental results are presented in the Results section, where a set of images is provided that compares the qualitative outcomes of the tests. In addition, quantitative data were collected and are presented in both tabular and graphical formats.

### 2.2. Numerical Model

**Figure 3:**
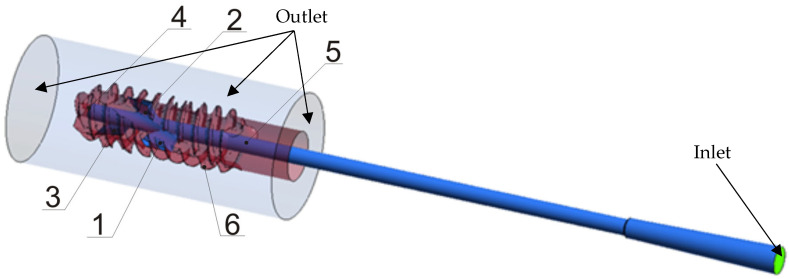
Fluid flow domain of the implant device 1–4—outlet holes (4 blocked if desired), 5—central feed channel, 6—thread (optional).

The transient numerical CFD simulations of the feeding of cement into a channel of the implant device model depicted in Figure 3 were performed using the Anasys Fluent 2024 R2 software. Taking into account the flow conditions and fluid properties, the flow was laminar. The pressure-based solver was used to solve the Navier–Stokes equations. It is common knowledge that the curing of PMMA is exothermic and that viscosity is temperature-dependent. In this study, the simulations are isothermal and confined to the initial workable period, prior to the primary exothermic peak. Experimentally, the temperature at the adapter/inlet was monitored using a PT100 sensor, MR-elektronika, Warsaw, Poland, while the surrounding medium was maintained at 36.6 ± 1 °C. During the injection interval represented in the CFD, no rapid rise in inlet temperature was observed. The isothermal conditions assumption was therefore justified.

As the implant channel was initially filled with air, the flow was simulated as two-phase (air–cement), with the application of the Volume of Fluid method, suitable for two separated phases. The Implicit formulation with a Volume Fraction Cutoff of 1 × 10^−8^ was utilized. The Sharp/Dispersed Interface model was considered in this study. Taking into account flow conditions (low flow velocity and relatively low pressure), air was considered incompressible. Preliminary tests using standard default parameters defined in Ansys Fluent for air caused solution instabilities. As was observed, high gradients of viscosity at air–cement phase interfaces could adversely influence the simulation convergence. According to technical support, the lower the ratio/gradient of the parameter at the interface, the more stable the solution is. It is claimed that at around a 1000-fold ratio, the second phase does not affect the flow in the first phase. Therefore, a decision was made to artificially overestimate the viscosity of air. After conducting several tests with task stability for different values, a decision was made to increase the viscosity of air a hundredfold, resulting in a ratio above 50,000, ensuring no impact on the cement flow.

The cement utilized in vertebral body augmentation procedures is a non-Newtonian fluid characterized by complex and time-dependent rheological properties. As demonstrated in previous experimental studies, its properties can be approximated by models suitable for shear-thinning fluids, supplemented with relations of the changes in its viscosity in time [27,28,29].

It is acknowledged that modeling cement as a Newtonian material constitutes a substantial simplification; however, a constant dynamic viscosity of 100 Pa·s was assumed. Within the scope of the present study, simulations were undertaken, and a comparison was made with a relatively short-term experiment. Therefore, the assumption of a constant viscosity over time may be justified. This simplification corresponds to the initial injection period when viscosity changes are still moderate, as reported in vertebroplasty rheology studies. We acknowledge that the model does not capture the pre-gel → post-gel transition. Furthermore, the flow velocity was found to be minimal, thereby reducing the impact of shear-thinning properties on the flow into individual holes in the model.

Based on our previous tests devoted to highly viscous fluids and multiphase flows in channels of complex shapes, we noted that using the fluid governed by shear-thinning and time-dependent behavior leads to significant problems with the simulation stability. It was hypothesized that the complex geometry of the distribution channels (i.e., the narrow active cross-section and the presence of threaded structures) in conjunction with the previously identified stability issues could have exacerbated and substantially extended the duration of the numerical data calculation. Following careful consideration, the decision was made to utilize the values for dynamic viscosity 100 Pa·s and density 1100 kg/m^3^, which had been determined in earlier experiments [28].

#### 2.2.1. Boundary Conditions

The inlet velocity boundary condition was applied at the entrance of the central feed channel. In the initial phase of flow, analogous to the syringe piston movement acceleration phase, a linear increase in the velocity of cement was defined. From t = 3.12 s onwards, the velocity remained constant at 1 mm/s. This velocity value corresponded to the volume flow rate delivered by the syringe during the experimental tests, where the plunger motion was controlled by the workstation to maintain a constant 1 mm/s piston velocity and thus a repeatable injection rate. On the transparent cylindrical surfaces of the domain opposite the inlet (See Figure 3), an outlet boundary condition was imposed with a constant pressure of 4 kPa gauge pressure. On the remaining surfaces—the supply channel, the openings, and the thread of the device—a no-slip wall condition was assumed.

#### 2.2.2. Numerical Model Discretization

**Figure 4:**
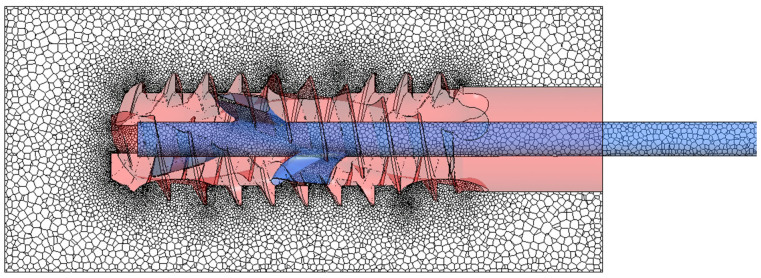
Mesh refinement in the cross-section plane along the channel axis.

The model was discretized using the Meshing module in Fluent 2024 R2 software. The polyhedron mesh type was selected to reduce the number of mesh control volumes when compared to a tetrahedral mesh while maintaining the quality of surface mapping. The number of control volumes directly impacts the time taken for the calculations to be performed. For time-varying calculations, limiting the mesh size is particularly important, as a system of equations must be solved iteratively for each control volume for each time step. The prepared meshes were refined in the channel constrictions and on the thread surface, as illustrated in Figure 4. Depending on the variant studied, there were approximately 1.9–2.1 million mesh nodes for the model without threads and almost 7 million for models with threads. Tests were carried out to determine mesh refinement level guaranteeing solution independence, as well as to assess the quality of the meshes using the following measures in Fluent Meshing: Minimum Orthogonal Quality, Maximum Tangent Skewness Quality, and Maximum Aspect Ratio. It is worth mentioning that we performed additional mesh independence tests by activating the adaptive mesh scheme for the calculation phase. It means that the solver could modify the mesh in each iteration to reach the desired convergence level and/or quality of the elements. The tests showed that in our case, the adaptive mesh was coarser in relation to the original mesh in places where there were homogeneous phases (cement or modified air) and significantly refined only at the boundaries between phases. The tests resulted in a significant increase in calculation time due to the need to generate the mesh essentially at every time step, but did not produce better quality results. Therefore, it was decided to use the mesh densities selected before the mesh adaptation trials. Additionally, they met all the quality criteria recommended for polyhedral meshes.

#### 2.2.3. Simulation Conditions and Convergence Criteria

In the numerical studies presented, pressure–velocity coupling with a first-order implicit transient formulation was used in the simulations. Additionally, high-order term relaxation was employed to enhance the stability of the solution, given the significant density difference between the two flow phases. Stability control was achieved through velocity limiting treatment, with a maximum velocity of 5 m/s imposed. Permissible limiting values were set at 10 for the flow Courant Number and 200 for the Volume Fraction Courant Number. The task was initialized with a time step of 0.001 s, and an adaptive time step was applied using the multiphase-specific method. The task duration was set to 30 s, and the global Courant number was limited to 200. The calculation was limited to 50 iterations per time step, which guaranteed the good convergence of the governing equations. Physically, calculations for a single case on two connected workstations (with a total of 60 cores and 256 GB of RAM) took between 220 and 300 h, depending on the device variant.

### 2.3. Experimental Model

Experimental tests were conducted on models G1 and G2 in functional variant v.3. Implant device models featuring variant channels were fabricated by stereolithography (SLA) with colorless ‘clear’ resin.

**Figure 5:**
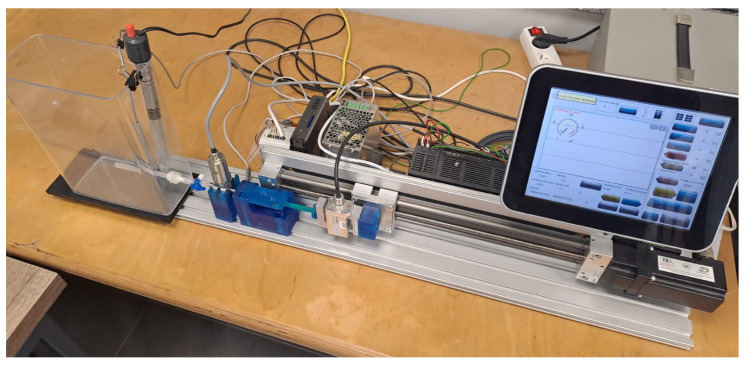
Experimental setup for cement injection testing.

The experimental tests were performed on a specialized laboratory workstation for flow testing that was developed at the LfC company, Zielona Góra, Poland [28], illustrated in Figure 5. Importantly, the workstation includes a guided plunger-driving unit that controls piston motion during injection. Therefore, cement was delivered using the workstation-controlled injection system rather than free-hand manual pushing. This workstation is equipped with an S-shaped strain gauge force sensor with a measuring range of ±1 kN, which is designed to measure compressive and tensile forces exerted on the syringe plunger. The built-in transducer provides an output signal of 0–10 V or 4–20 mA with a 24 V DC power supply.

A class A PT100 resistance sensor with a measuring range of −50 to 400 °C was used to measure the temperature of the medium and environment. The sensor is equipped with a 2 m cable that is resistant to temperatures of up to 400 °C, enabling stable measurements to be taken under the conditions of the experiment. The temperature sensor and pressure transducer were placed in an adapter located between the cement dispenser and the implant model. The pressure transducer was positioned directly inside the adapter connecting the syringe to the implant model, via a three-way channel with one connector free-used to vent the cement delivery line. Care was taken to keep the diameter constant in all connections. The adapter geometry and the sensor location were mapped to the CFD inlet plane to ensure consistency between experimental and numerical boundary conditions. The mapping also accounted for the slight increase in flow-path length introduced by the three-way valve.

A GEFRAN TPFAS series Miniature Flush Diaphragm Pressure Transmitter (MFDPT), Gefran S.p.A., Provaglio d’Iseo, Italy was used to measure pressure. It has a measuring range of 0 to 3 MPa, an accuracy of ±0.5% Full Scale Output, a response time of less than 1 ms, and an output signal of 4–20 mA. Its tightness corresponds to IP65. The measurement point was selected based on clinical procedure, i.e., to capture pressure at the location relevant to surgery associated with the highest risk of system leakage and unsafe force build-up during cement delivery.

The station was also equipped with a tank containing a liquid (demineralized water) at a constant temperature, regulated by a heater. The temperature was maintained at 36.6 ± 1 °C. This was to simulate the physiological conditions associated with human body temperature and interstitial pressure affecting the implant placed in the body.

The present study employed a commercially available injectable bone cement based on polymethyl methacrylate. This material is commonly used in vertebroplasty and was applied here as the medium. The composition of the powder component comprises a mixture of polymethyl methacrylate polymer, methyl methacrylate, and methyl acrylate copolymer. An inorganic contrast agent, zirconium dioxide, is also included, along with a polymerization initiator, benzoyl peroxide. The liquid part of the mixture includes methyl methacrylate monomer, a polymerization accelerator in the form of N,N-dimethyl-p-toluidine, and a stabilizer—hydroquinone. The cement was prepared in accordance with the manufacturer’s recommendations and instructions, because the proportions of the components are important for the properties of cements during and after implantation [30,31]. Following the blending of the two components, the cement takes a fluid consistency and then can be applied using a syringe. For each test, three 3-cm^3^ syringes of the material under investigation were prepared, which were successively connected during injection to the inlet hole of the adapter. Each injection was carried out under workstation-controlled piston motion to keep the delivery rate consistent between tests. During the administration of the medium, the force exerted on the syringe plunger, the applied pressure, and the temperature of the liquid surrounding the implant model were measured. The experiment was conducted at a liquid temperature of 36.6 °C ± 1 °C.

In the experimental method, the volume of cement introduced into the canals was determined based on the geometry of the syringe and the displacement of the plunger. The speed of piston motion equal to 1 mm/s used in the experiment corresponds to the values used in clinical conditions during PMMA cement injection in vertebroplasty/kyphoplasty and during screw stabilization. In clinical practice, the aim is to administer the cement slowly and in a controlled manner, while monitoring its spread using X-ray imaging to reduce the risk of leakage. In addition, this speed allows the full prepared dose of cement to be administered before it polymerizes.

At a rate of 1 mm/s, the internal diameter of the system enables the reliable measurement of force and pressure. Furthermore, it has been demonstrated that this method serves to mitigate the impact of variability in cement viscosity, thereby enhancing the repeatability of the process. Increased velocities have been observed to result in the occurrence of leakage or disruption to the flow. It is therefore recommended that a velocity of 1 mm/s be employed in order to ensure consistent and repeatable delivery within the experimental setup.

## 3. Results

The section containing the results has been divided into two parts, with qualitative and quantitative comparisons of numerical and experimental data presented.

### 3.1. Qualitative Comparison of Results

A qualitative comparison of the results of numerical and experimental studies confirms the reliability of the adopted research methodology. The results of numerical tests were verified in experimental tests for version v.3 and design G1 with perpendicular side channels and G2 with side channels located at an angle of 70° to the central channel.

**Figure 6:**
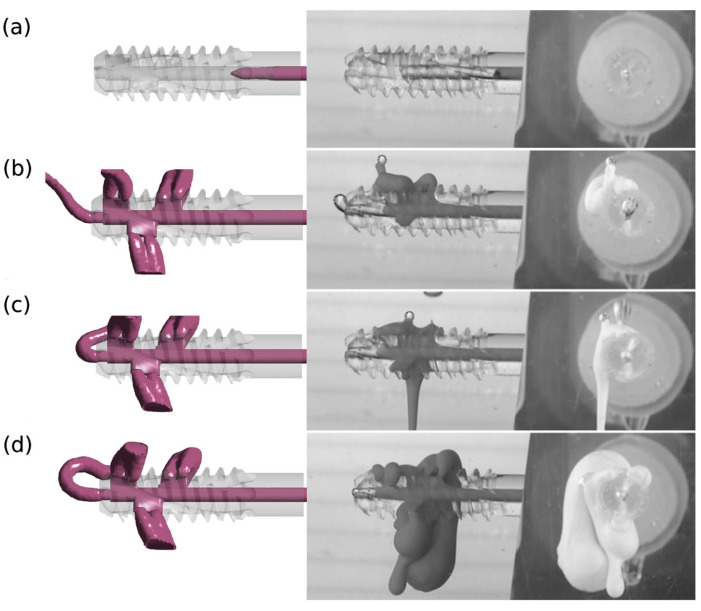
Injection of bone cement at different time points: (**a**) 5 s, (**b**) 10 s, (**c**) 20 s, (**d**) 30 s (measured from the time when the cement reached the inlet of the implant device) for the case G1 v.3. Results obtained using the numerical method (left) and the experimental method (right).

**Figure 7:**
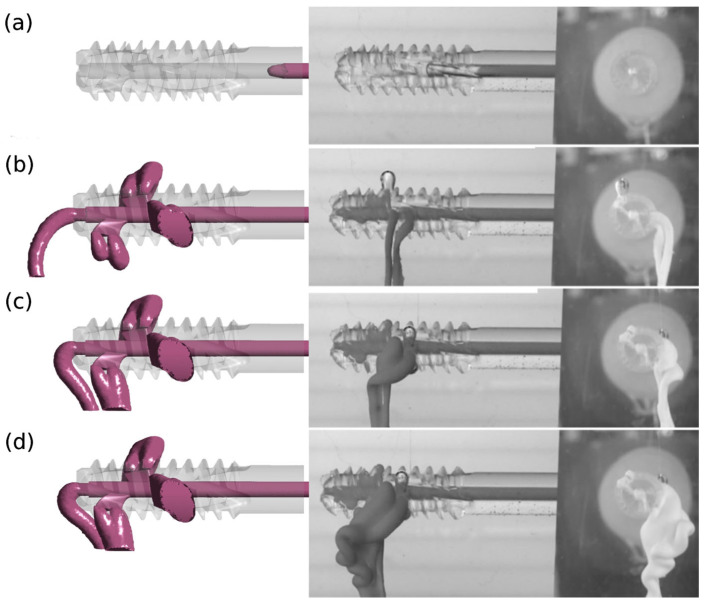
Injection of bone cement at different time points: (**a**) 5 s, (**b**) 10 s, (**c**) 20 s, (**d**) 30 s (measured from the time when the cement reached the inlet of the implant device) for the case G2 v.3. Results obtained using the numerical method (left) and the experimental method (right).

Figure 6 and Figure 7 illustrate images from numerical and experimental studies conducted at designated injection times. In numerical studies, the model periosteal medium was characterized by constant parameters, while in laboratory studies, functional bone cement changed its properties over time. The simulation and control of viscosity variability under these conditions is challenging, and this poses a significant challenge during cement preparation and application [32]. Despite these limitations, concerted efforts were made to conduct the experiment in such a manner that the duration was kept to a minimum and the condition of viscosity stability was met.

The divergent images obtained from both studies suggest the impact of this factor, particularly evident in the case of cement flow through the central channel (4) for G1 v.3 and G2 v.3. Moreover, the presence of randomly distributed air bubbles in the bone cement has been observed to influence the imaging outcomes of flow through individual holes.

### 3.2. Quantitative Numerical Simulation Results

The tables below provide a concise summary of the results of the numerical calculations. The data presented herein demonstrate the volume of medium that is discharging from individual outlet holes (1 ÷ 4 in Figure 1), in addition to the pressure within these holes, as computed in the final second of the cement injection process.

A comparison was made between designs G1 and G2 in version v.1. This comparison revealed that the angle of inclination of the side channels relative to the central channel was reduced from 90° to 70°. This change resulted in a decrease in the amount of cement flowing out of opening (1) by 8.6% points. Concurrently, an increase was recorded in the flow of cement through the side openings (2) and (3) by 0.6% points and 2.4% points, respectively, and through the central opening (4) by 5.6% points (see Table 2 for details). The modification of the angle of the side channels also affected the pressure in the discharge openings (see Table 3). The pressure for the G2 v.1 design in the central hole (4) increased by 10% points, while in the side holes (1), (2), (3) it was lower by 12.3% points, 7.1% points, and 3.4% points, respectively, compared to the G1 v.1 design. The volume of cement flowing out for case G2 v.1 in relation to G1 v.1 decreased by 4.2%.

In both G1 and G2 designs in version v.2, the additional blocking of the central channel naturally increased and changed the distribution of the volume of cement flowing through the side channels (see Table 4).

In the case of the G2 v.2 design, which features inclined side channels at a 70° angle, there was a 6.8% point decrease in medium flow out for outlet (1) when compared to the G1 v.2 design. Conversely, there was an increase in medium flow out for the other two outlets: 2.6% points for outlet (2) and 4.2% points for outlet (3). The results indicate that the flow of medium in the G2 implant is more uniform when the central channel (4) is blocked.

The configuration of the side channels in the G2 design also exerts a favorable influence on the reduction in pressure in the inlet/feed hole and the outlet holes (1 ÷ 3) in the event of obstruction of the central hole (4). The medium pressure in the G2 design was found to be 15.4% lower for the side opening (1), 7.8% lower for (2), and 3.9% lower for (3), respectively. The data are presented in Table 5.

The implementation of a threaded configuration in the lateral outlet region also influenced the quantity and distribution of the medium surrounding the implant model (see Table 6). In the case of the G1 design in version v.3, a marked decrease in the quantity of medium dischargingthrough the side hole (1) was observed, accompanied by an increase in the amount of medium flowing out of the central hole (4) when compared to the non-threaded version v.1. For both G1 and G2 (v.3) designs, the variation in the volume of medium flowing out of the individual holes did not exceed 1.5% points. It can be concluded that similar results were obtained for G1 and G2.

The pressure results in the individual outlet holes being significantly higher for the v.3 design, with the side holes covered by the thread. The G2 v.3 design, characterized by inclined channels at an angle of 70°, exhibited higher pressures compared to the G1 v.3 model (see Table 7 for details). For G2, the pressure at the outlet of the central channel (4) was found to be 5.5% higher, and for the outlet holes (1), (2), and (3), the increases were 101.8%, 84.1%, and 52.5%, respectively. In this case, large pressure differences may be affected by various overshadowing factors, and thus by changes in the surface area of the holes due to the thread outline on the outer surface of the implant. This is also reflected in the approximately fivefold difference in pressure values for implants without (v.1 and v.2) and with threads (v.3).

**Figure 8:**
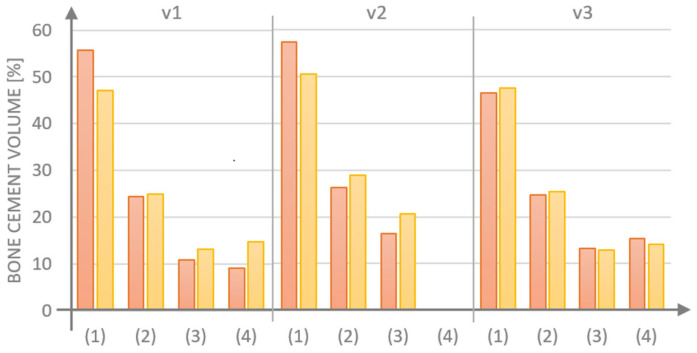
Summary of the amount of medium flowing out through individual holes in implant models G1 (red) and G2 (yellow) tested in three design variants.

For each model that was examined, the majority of cement was observed to dischargingthrough the side hole (1), with the least amount of cement being released through hole (3), which was positioned the furthest from the injection site (Figure 8). The obstruction of the central hole (4) resulted in increases in cement volume for G1 (v.1 vs. v.2) of 1.6% for hole (1), 1.8% for opening (2), and 5.7% for opening (3). In contrast, for G2 (v.1 vs. v.2), the cement volume increased by 3.4% for opening (1), 3.9% for hole (2), and 7.5% for hole (3). The integration of a threaded variant v.3, which obstructs the flow inthe side holes, resulted in a more favorable distribution of the medium for the G1 design with perpendicular side channels.

**Figure 9:**
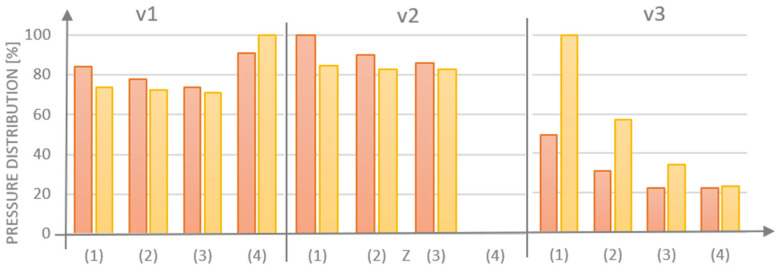
Comparison of pressure distribution in individual inlet and outlet openings in G1 (red) and G2 (yellow) implant models tested in three design variants.

Figure 9 illustrates the full range of pressure distribuiton derived from the numerical analysis. For the v.1 models, the pressure in the inlet openings (0) was calculated as 303,439 Pa for G1 and 315,075 Pa for G2. In the side outlet holes (1 ÷ 3), the range was from 4134 Pa to 4889 Pa, values that approximate the assigned boundary condition. In the central hole (4), the pressure was found to be higher, with measurements of 5271 Pa for G1 and 5806 Pa for G2. It was hypothesized that clogging of the central opening (4) in version v.2 would cause an increase in pressure in the inlet opening. However, no significant effect on the pressure change in the side openings (1 ÷ 3) was observed in the case of model v.3, where the thread partially obscures the side outlet holes.

### 3.3. Experimental Results

The objective of this part of the study was to investigate the delivery of cement under experimental conditions for the specified bone cement in channels selected on the basis of numerical analyses. Preliminary assessments were facilitated by numerical investigations, which enabled the estimation of pressure values and the flow pattern through holes in the implanted device. The investigation revealed that the flow pattern was most favorable to geometric models G1 v.3 and G2 v.3. Due to the limited resources of cement samples for testing, at the current preliminary stage of preparation for implant development, two studies were conducted, one for each of the selected channel geometries.

The channels were manufactured using stereolithography technology from transparent resin, employing a 3D printer (Form 3B+, Formlabs) and mounted in the LfC company’s research station. The experiments were conducted using a procedure that, in principle, could be employed in an operating room setting during cementoplasty surgery. The assumption was made that the mean volume of cement administered during the procedure would be 6 cm^3^ (6 mL) [1,9]. The administration of the substance will take place in three consecutive injections, with each injection being 3 cm^3^. The first syringe is used to fill the delivery system together with the adapter, which is equipped with a pressure transducer. The injections were administered using disposable syringes that are specifically designed for vertebroplasty procedures. The initiation of the experiment was designated as the moment (t_0_ = 0 s) at which, in accordance with the manufacturer’s guidelines, the cement components initially came into contact with each other.

The powder phase was dispensed into an 80 cm^3^ syringe; next, the liquid phase was added. Subsequent to this, the two phases were mixed by means of a vigorous shaking process, which was continued for a period of approximately 20 s. Following the mixing and attainment of a “thin dough” consistency in accordance with the manufacturer’s guidelines, three working syringes were filled. At t_1_ = 50 s, after connection of the first syringe with a Luer-lock connector, the introduction of cement into the channel commenced. At this time, the application of the first dose proceeded without any measurable increase in pressure or force. At t_2_ = 120 s, a second syringe was connected.

**Figure 10:**
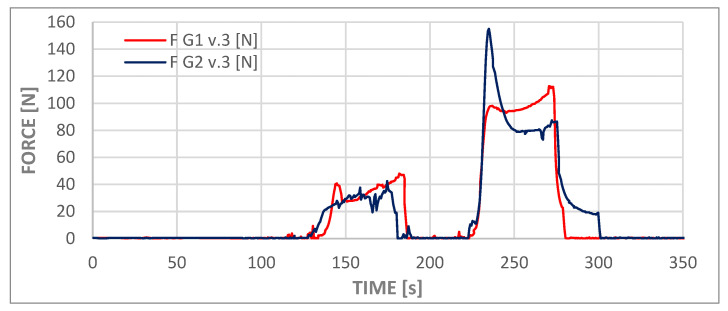
Experimental curves of injection force versus time for cement delivery in variants G1 v.3 and G2 v.3.

**Figure 11:**
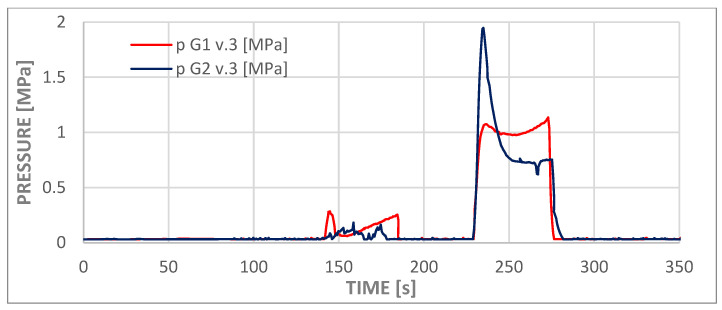
Experimental curves of injection pressure versus time for cement delivery in variants G1 v.3 and G2 v.3.

The process of administering the contents of the second syringe was recorded by force and pressure sensors and is visible in graphs in Figure 10 and Figure 11 as the first deviations from values close to zero on both graphs (from approximately t = 145 s to t = 180 s). This phase of the experiment was followed by a second syringe replacement and the injection of the third portion of cement (from approximately t = 230 s to t = 275 s). The third phase of administration corresponded to the flow that was analyzed in the numerical simulations. At this stage of the experiment, the viscosity of the cement was comparable to that employed in the numerical simulations. Consequently, the measured pressure values fell within the ranges presented in Table 7 for both models, slightly above 1.4 MPa. The pressure measurement at the test station was performed at a location analogous to the inlet in the numerical model. The cement dispenser, in the form of a syringe, is connected to the adapter inlet, to which a pressure transducer and a temperature sensor are connected. The adapter output is connected to a T-piece, which allows the adapter to be vented while filling the measuring system with cement and acts as a barrier to the water filling the implant model.

In the course of these experiments, the force exerted on the syringe piston was found to be less than 120 N in the case of variant G1 v.3, whereas for variant G2 v.3, the instantaneous force exerted was approximately 30% higher, reaching 155 N.

The discrepancies observed in the pressure values obtained from numerical simulations and experiments are considerable; however, due to the substantial expense associated with the preparation method and the fabrication of the channels, it was not anticipated to conduct a large number of tests. It can be hypothesized that the averaged results of experimental studies would be more closely aligned with numerical simulations.

The divergent pressure curves observed for the second and third phases, as depicted in both curves in the graph, suggest that the chemical processes involved in polymer setting may vary significantly among different samples, despite the repetition of external conditions such as cement temperature, ambient temperature, and water temperature. The elevated peak observed in the G2.v3 test may be indicative of two potential phenomena. Firstly, the formation of a cement plug at the tip of the syringe, and secondly, the overcoming of high friction forces by the system during the initial piston movement.

## 4. Conclusions

- A series of numerical and experimental studies was conducted on the flow of cement during its delivery through a spinal implant. It should be emphasized that the comparison of results was made qualitatively, based on general trends in both parts of the study, rather than by comparing quantitative parameters.
- The observed discrepancies in experimental studies for identical structural solutions are attributable to alterations in the properties of bone cement over time and the probable effect of cement aeration as a consequence of the mixing of components.
- The configuration and inclination of the transport channels employed for delivering bone cement to the implantation site exert a significant influence on the delivery parameters (i.e., pressure, force exerted on the syringe plunger) and the distribution of bone cement. Nevertheless, it should be emphasized that interaction with bone tissue and the phenomenon of backpressure were not taken into account in our research.
- The utilization of side outlet holes at an angle of 70° has been shown to favorably influence the administration pressure and the medium flow, resulting in acceptable cement distribution around the implant and reducing injection pressure.
- It has been demonstrated that obstructing the central channel during the installation process does not lead to a substantial increase in pressure; however, it exerts a favorable influence on the flow of the medium through the lateral outlet holes into the immediate vicinity of the implant.
- The incorporation of thread in the vicinity of the outlet holes exerts a substantial influence on the medium distribution, leading to an increase in the pressure of cement delivery, particularly in the context of angled outlet holes.
- Notwithstanding the favorable conditions anticipated for the predicted distribution of cement around the implant, elevated delivery pressure may pose a significant risk to the safety of the procedure by reducing control of the injection and cement distribution in the surrounding bone tissue. This consideration must be incorporated into the design process.
- It should be noted that the current stage of research is a preliminary analysis, deliberately conducted under reduced resistance conditions to enable unambiguous comparative validation.

## 5. Summary

This publication presents the dynamic viscosity, handling characteristics, and injection methods of PMMA bone cements used for bone-filling procedures, including various techniques and different types of surgical procedures, such as vertebroplasty, kyphoplasty, and stentoplasty. The study examined a novel system for administering cement with anchorage in bone and between bones. A two-stage study was conducted. The objective of the numerical simulations was to determine the pressure within the system, the uniformity of cement flow, the influence of the outlet hole positions, and the angle of flow from the primary channel. The experimental studies confirmed the simulation results and verified the feasibility of delivering cement in batches using three syringes. Subsequent investigation will extend the rheological model of cement to encompass alterations in viscosity over time, whilst incorporating the trabecular structure adjacent to the implant.

In terms of experimental research, the following considerations are being given thought: firstly, the inclusion of an outline of the vertebral body model; secondly, the use of artificial bone material to account for resistance in conditions similar to real-life conditions. Furthermore, the future study will involve conducting more experiments using cements from different manufacturers and increasing the number of samples in order to obtain information on the repeatability of cement application through an implant into the bone. Additionally, we consider that a more detailed parametric analysis of local outlet obstruction will be addressed in future work.

When it comes to the possibility of expanding the CFD modeling, two separate approaches are considered. The first option involves creating a sequence of numerical analyses of cement flow with constant but varied parameters (viscosity and shear rate). The subsequent investigation will extend the rheological model of cement to include time-dependent viscosity evolution during curing (pre-gel → post-gel) and non-Newtonian features, synchronizing CFD with the experimental injection timeline to enable time-resolved validation of pressure and force.

## Figures and Tables

**Table 1:** Comparison of dynamic viscosity, handling characteristics, and injection methods of PMMA bone cements.

Brand	Viscosity (Manuf.)	Viscosity (Start) [Pa·s]	Viscosity (End Stage) [Pa·s]	Working Time [min]	Hardenning Time [min]	Injection Method	Sources
Osteopal V	Low viscosity	200–400	1500–1600	8–10	12–14	Manual injection, syringe/cannula (4.2 mm diameter)	[19,20,21]
Mendec	Standard viscosity	100–200	370–470	3–4	7–8	Manual injection, standard PVP cannula	[20]
Vertecem V+	High viscosity	200–400	1500–1600	3–4	7–8	Manual injection, transpedicular cannula	[20,23]
Vertecem Synthes	Low viscosity	46 ± 15	~55	Viscosity increases within 0.5 min		Injection with the instrumented syringe	[25]
Simplex P	Medium viscosity	100–200	370–470	3–6	7–15	Manual injection, cement gun, transpedicular PVP	[21,26]
PMMA Synthes	Low viscosity	20–200	200–2000	3–6	10–15	Manual injection syringe, biopsy needle	[22]
Confidence Spinal Cement System	High viscosity	~1000	~1000	8–10	8–12	Manual injection, biopsy needle	[24]
Cohesion Bone Cement	Medium viscosity	~350	~350	~26	>20	Manual injection, syringe/cannula (2.8 mm diameter)	[24]

**Table 2:** Percentage volume of medium discharging through individual outlet holes in structural models G1 and G2 (version v.1) with different angles of inclination of the side.

	G1 v.1		G2 v.1	
	V [mm^3^]	V [%]	V [mm^3^]	V [%]
outlet side hole 1	0.738	55.70	0.598	47.12
outlet side hole 2	0.323	24.38	0.317	24.98
outlet side hole 3	0.143	10.79	0.167	13.16
outlet central hole 4	0.121	9.13	0.187	14.74
Total Volume	1.325	100.00	1.269	100.00

**Table 3:** Fluid pressure recorded in the last second of injection at the inlet and outlet openings for models G1 and G2 (version v.1) with different angles of inclination of the side channels.

	Pressure [Pa]		Relative Difference [%]
	G1 v.1	G2 v.1	
outlet side hole 1	4889	4287	−12.3
outlet side hole 2	4528	4206	−7.1
outlet side hole 3	4279	4134	−3.4
outlet central hole 4	5271	5806	10.1
inlet section	303,439	315,075	3.8
section located 5 mm downstream the inlet	271,235	281,539	3.8

**Table 4:** Percentage volume of medium discharging through individual outlet holes in structural models G1 and G2 (version v.2) with different angles of inclination of side channels.

	G1 v.2		G2 v.2	
	V [mm^3^]	V [%]	V [mm^3^]	V [%]
outlet side hole 1	0.766	57.34	0.646	50.51
outlet side hole 2	0.350	26.20	0.369	28.85
outlet side hole 3	0.220	16.47	0.264	20.64
outlet central hole 4	-	-	-	-
Total Volume	1.336	100.00	1.279	100.00

**Table 5:** Fluid pressure recorded in the last second of injection at the inlet and outlet openings for models G1 and G2 (version v.2) with different angles of inclination of the side channels.

	Pressure [Pa]		Relative Difference [%]
	G1 v.2	G2 v.2	
outlet side hole 1	5045	4269	−15.4
outlet side hole 2	4531	4179	−7.8
outlet side hole 3	4340	4172	−3.9
outlet central hole 4	-	-	-
inlet section	331,369	319,824	−3.5
section located 5 mm downstream the inlet	299,253	285,492	−4.6

**Table 6:** Percentage volume of medium dischargingthrough individual outlet holes in structural models G1 and G2 (version v.3) with different angles of inclination of side channels.

	G1 v.3		G2 v.3	
	V [mm^3^]	V [%]	V [mm^3^]	V [%]
outlet side hole 1	0.61	46.54	0.64	47.55
outlet side hole 2	0.32	24.73	0.34	25.40
outlet side hole 3	0.17	13.29	0.17	12.85
outlet central hole 4	0.20	15.44	0.19	14.20
Total Volume	1.30	100.00	1.34	100.00

**Table 7:** Fluid pressure recorded in the last second of injection at the inlet and outlet openings for models G1 and G2 (version v.3) with different angles of inclination of the side channels relative to the central channel.

	Pressure [Pa]		Relative Difference [%]
	G1 v.3	G2 v.3	
outlet side hole 1	18,518	37,361	101.8
outlet side hole 2	11,619	21,388	84.1
outlet side hole 3	8403	12,818	52.5
outlet central hole 4	8311	8768	5.5
inlet section	1,433,770	1,452,830	1.3
section located 5 mm downstream the inlet	1,418,719	1,437,735	1.3

## Data Availability

The original contributions presented in this study are included in the article. Further inquiries can be directed to the corresponding author.
